# Myocardial fibrosis is associated with worse left ventricular strain and synchrony assessed by novel CMR imaging in the general population: data from the Akershus Cardiac examination 1950 study

**DOI:** 10.1007/s10554-026-03746-1

**Published:** 2026-06-19

**Authors:** Thakshani Wimalanathan, Joanna Sulkowska, Aklilu Woldegabriel Melles, Michael Fredrik Paus, Julia Brox Skranes, Trygve Berge, Arnljot Tveit, Helge Røsjø, Torbjørn Omland, Magnus Nakrem Lyngbakken, Siri Lagethon Heck

**Affiliations:** 1 Department of Diagnostic Imaging, Division of Diagnostics and Technology, Akershus University Hospital, Lørenskog, Norway; 2 K.G. Jebsen Center for Cardiac Biomarkers, Institute of Clinical Medicine, Faculty of Medicine, University of Oslo, Oslo, Norway; 3 Department of Cardiology, Division of Medicine, Akershus University Hospital, Lørenskog, Norway; 4 Department of Medical Research, Bærum Hospital, Vestre Viken Hospital Trust, Gjettum, Norway; 5 Institute of Clinical Medicine, Faculty of Medicine, University of Oslo, Oslo, Norway; 6 Akershus Clinical Research Center (ACR), Division of Research and Innovation, Akershus University Hospital, Lørenskog, Norway

**Keywords:** Extracellular volume fraction, Mechanical dispersion myocardial fibrosis, Myocardial strain, Myocardial synchrony, CMR feature tracking, Fast strain-encoded MR (fSENC)

## Abstract

Investigate the relationship between myocardial fibrosis and left ventricular (LV) deformation and mechanical synchrony using state-of-the-art cardiac magnetic resonance imaging (CMR) in an elderly general population. In 200 participants without known coronary artery disease, all born in 1950, diffuse fibrosis was quantified by septal extracellular volume (ECV) fraction and focal fibrosis by late gadolinium enhancement (LGE). LV global longitudinal and circumferential strain (GLS, GCS) and mechanical dispersion (MD) were derived by CMR-feature tracking (CMR-FT). Myocardial deformation was also assessed using fast strain-encoded MR (fSENC) percentage of LV segments with normal longitudinal and circumferential strain (≤ -17%). The associations between myocardial fibrosis and bothstrain and mechanical dispersion were examined using regression analyses. Median age was 69.0 (interquartile range (IQR) 68.6–69.3) years and 52% were male. In adjusted models, LGE presence and volume were associated with worse GLS (presence: β = 0.83, 95% CI 0.02 to 1.63; volume: β = 0.17, 95% CI 0.04 to 0.30) and lower fSENC % normal myocardium (presence: β = -6.68, 95% CI -13.33 to -0.04; volume: β = -1.66, 95% CI -3.14 to -0.18). The association between LGE (presence and volume) and GCS was attenuated when adjusting for indexed LV end diastolic volume. ECV values above 26% were associated with worse GLS, GCS and lower fSENC % normal myocardium. Participants with focal scars had significantly higher median MD (89.0 ms (IQR 71.2, 121.1) vs 78.0 ms (IQR 60.4, 97.3)), and focal scars were associated with MD above the sex specific upper quartile (OR 2.64, 95% CI (1.18, 5.94) for LGE presence and 1.22, 95% CI (1.05, 1.42) for LGE volume). In this elderly general population, focal myocardial fibrosis was associated with worse LV deformation and synchrony. There was a threshold effect with a non-linear association between diffuse fibrosis and strain.

**Graphical abstract:**

**Figure:**
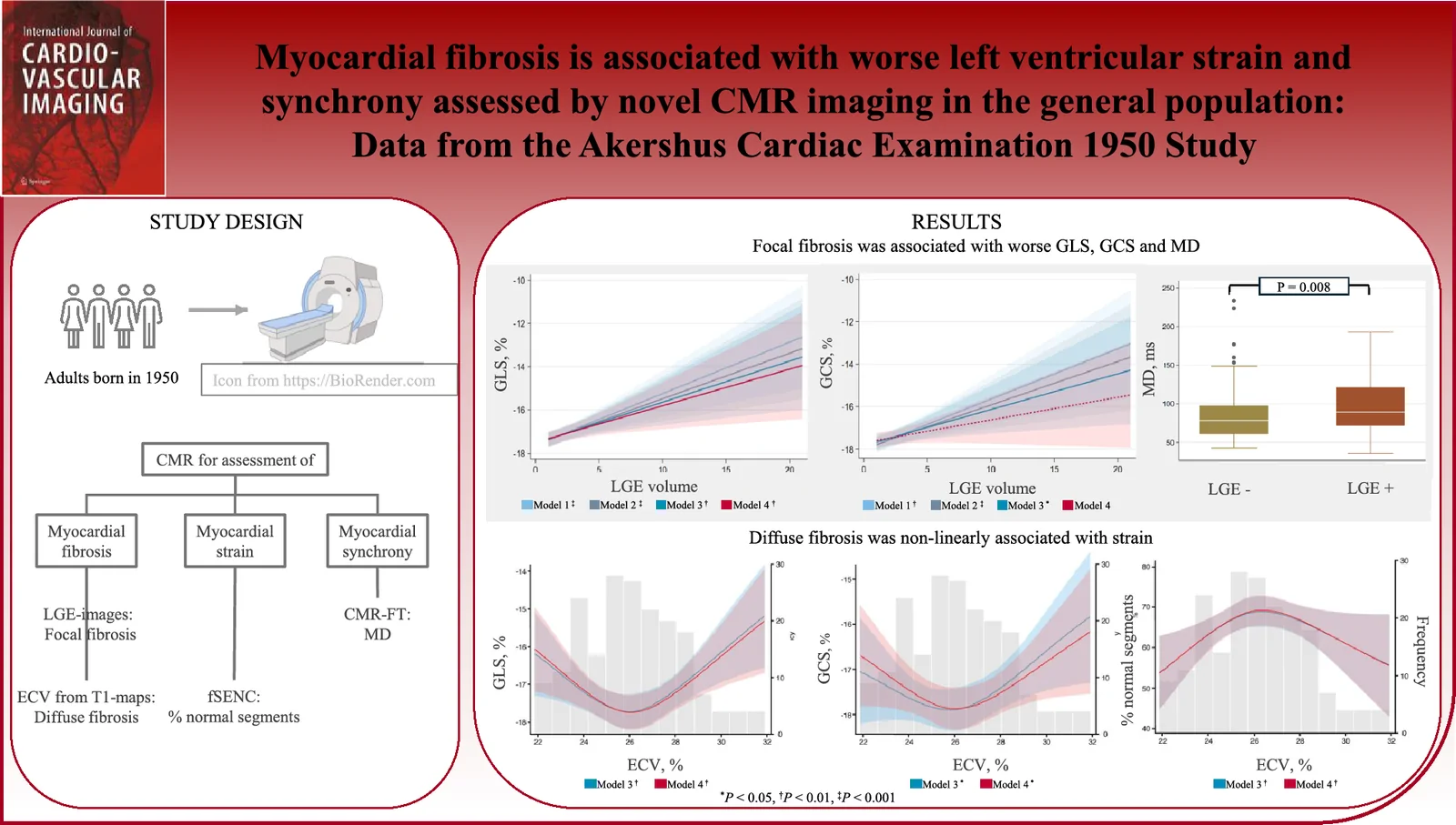

**Supplementary Information:**

The online version contains supplementary material available at https://doi.org/10.1007/s10554-026-03746-1.

## Background

Myocardial fibrosis is a common characteristic of many cardiovascular diseases [1], yet it can also be observed among individuals without clinically apparent disease [2, 3]. The presence of myocardial fibrosis is associated with increased risk of adverse cardiovascular events in the general population [2, 4], suggesting that it may represent a key step in the development of overt cardiovascular pathology [1]. The distribution pattern of myocardial fibrosis varies, reflecting distinct underlying mechanisms. Focal fibrosis typically results from collagen replacement in areas of localized cardiomyocyte necrosis, causing focal scarring. In contrast, in diffuse myocardial fibrosis, collagen accumulates diffusely in the extracellular matrix throughout the myocardium [5]. This diffuse pattern is present in nearly all chronic cardiac diseases, many systemic diseases, and is also associated with aging [1]. Cardiac magnetic resonance (CMR) enables assessment of focal fibrosis by well-established late gadolinium enhancement (LGE) sequences [6] and of diffuse fibrosis by calculation of the extracellular volume fraction (ECV) from T1 mapping techniques [7].

Recent developments in clinical image post-processing software have introduced novel metrics of myocardial mechanics, which are emerging as tools for assessing cardiac function. Myocardial strain is a measure of the relative myocardial deformation and mechanical dispersion (MD) reflects the contraction synchrony among the left ventricular (LV) segments. Both strain and MD have diagnostic and prognostic value across a wide range of cardiac diseases when assessed by echocardiography [8, 9].

Although most commonly acquired by echocardiography, these metrics can also be extracted by CMR, which provides detailed and reliable characterization of cardiac structure and function. Feature tracking (CMR-FT) traces myocardial motion on conventional cine sequences, enabling the quantification of strain measurements, while also enabling assessment of MD. Despite being a relatively established measure in echocardiography, MD has been less explored in CMR and lacks a standard methodology. Fast strain-encoded MR (fSENC) is an advanced novel CMR tagging technique where color-coded maps are generated from fast, single shot sequences encoding strain in a single heartbeat for each plane in a through-plane direction. The percentage of segments with normal deformation (fSENC% normal myocardium) is reported to be a sensitive tool for the detection of subclinical LV dysfunction during cardiotoxic chemotherapy and in stage B heart failure [10, 11]. Assessment of strain by both feature tracking and fSENC has demonstrated excellent reproducibility [12].

Uniquely, CMR permits simultaneous assessment of focal and diffuse myocardial fibrosis and subtle functional myocardial abnormalities.

Associations between extent of myocardial fibrosis and both worse strain and increased MD have been demonstrated across different cardiac disease states like myocardial infarction, heart failure, hypertrophic cardiomyopathy, myocarditis and systemic sclerosis [13–17]. However, subclinical alterations may occur prior to overt disease, and there is little data available on these associations assessed by CMR in the general population without established coronary artery disease. Therefore, the aim of the current study is to investigate whether and how the extent of myocardial fibrosis may influence the mechanics of the LV assessed by novel CMR techniques in an elderly general population.

## Methods

### Study overview and study population

**Fig. 1:**
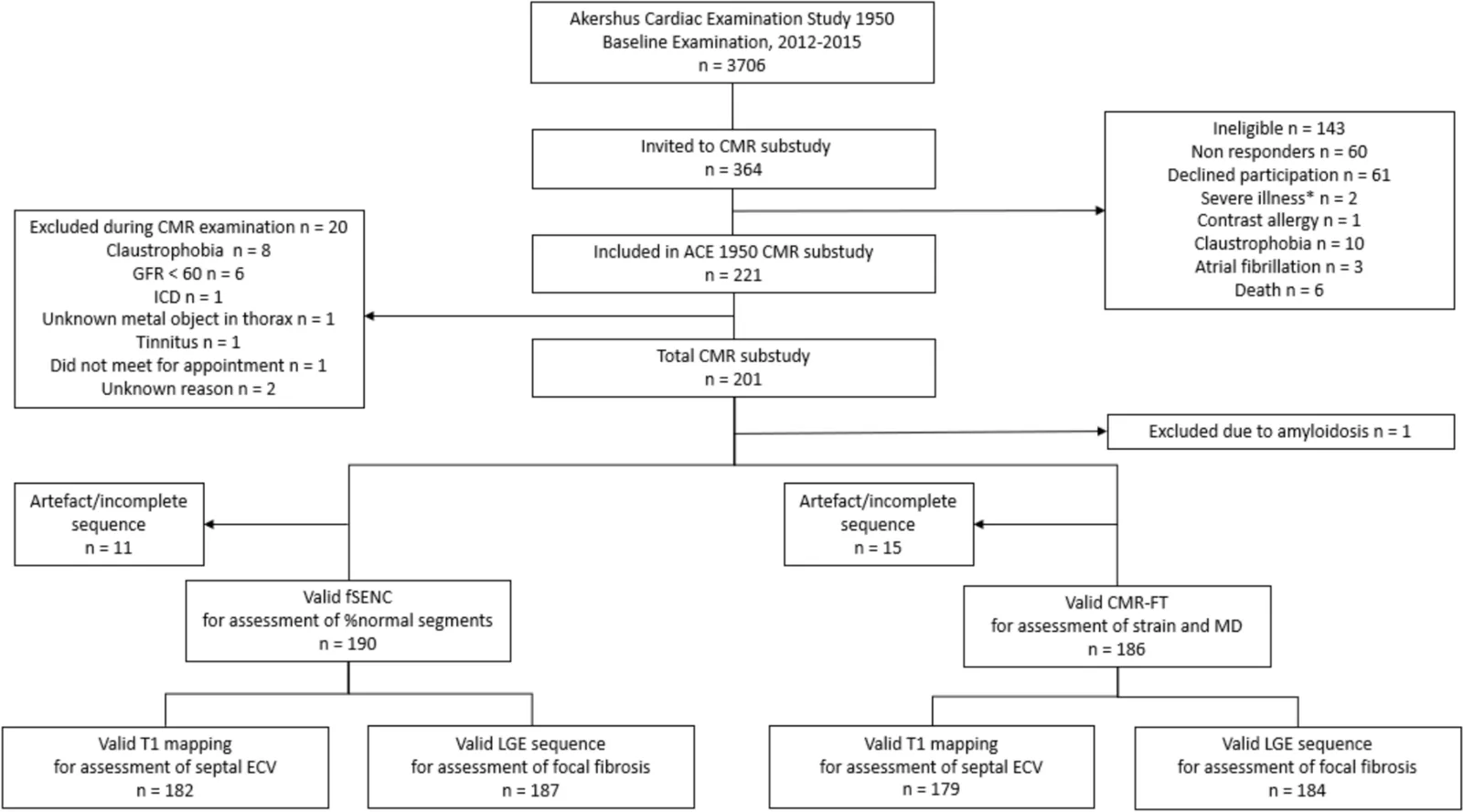
Flow chart of study population. ACE, Akershus Cardiac Examination; CMR, cardiac magnetic resonance; ECV, extracellular volume; fSENC, fast strain encoded magnetic resonance; GFR, glomerular filtration rate; ICD, implantable cardioverter defibrillator; LGE, late gadolinium enhancement.MD, mechanical dispersion

The Akershus Cardiac Examination (ACE) 1950 Study is a prospective study of 3706 community-dwellers of both sexes born in 1950 and residing in Akershus county. For the current CMR substudy, 364 participants with the highest and lowest baseline concentrations of cardiac troponins were invited to enrich the cohort with low- and high cardiovascular (CV) risk participants. Participants were included if they had preserved renal function (estimated glomerular filtration rate [eGFR] > 60 mL/min/1,73m^2^) and no known coronary artery disease (CAD) at the ACE 1950 Study baseline. In total, 201 of these participants underwent CMR examination (Fig. 1). One participant was excluded after CMR acquisition due to findings suggestive of amyloidosis. Baseline examinations were conducted between 2012–2015 and CMR was performed between 2018–2019, and the median time from baseline to CMR was 58 (interquartile range [IQR] 53–63) months. Both the ACE 1950 study and the CMR substudy have been described in detail previously [18, 19].

### Biochemical analyses

Routine clinical chemistry was performed within a week before CMR. Hematocrit was measured at the time of CMR.

### CMR protocol and assessment

A 1.5-T MRI scanner (Achieva; Philips Medical Systems, Best, The Netherlands) was used to perform all CMR examinations, and image analyses were performed according to Society for Cardiovascular Magnetic Resonance (SCMR) guidelines on dedicated image post-processing software (CVI42, v5.13.5, Circle Cardiovascular Inc, Calgary, Canada) [7, 20]. All examinations included steady-state-free-precession sequences (SSFP) with 8 mm short-axis slices covering the ventricles and three standard long-axis views. Temporal resolution was 30 frames per cardiac cycle. Global longitudinal (GLS) and global circumferential strain (GCS), and segmental time to peak longitudinal strain were derived from these sequences using two-dimensional CMR-FT. GLS and segmental time to peak longitudinal strain were obtained from three long-axis views, and GCS from the short-axis stack. MD was calculated as the standard deviation of the time to peak longitudinal strain of the 16 American Heart Association (AHA) segments and adjusted for heart rate prior to the analysis. MD was corrected for heart rate by dividing the participant’s MD by heart rate and multiplying by 60 (normalized to a heart rate of 60 beats per minute), as differences in cardiac cycle length may influence time to maximal contraction. In-depth descriptions of the strain and MD assessments have previously been provided [21]. All feature tracking and MD analyses were performed by JS.

SSFP sequences were also used to quantify LV volumes, ejection fraction (EF), and mass. Trabeculations and papillary muscles were included in LV volume and excluded from LV mass estimation.

The CMR protocol included fSENC sequences in three long axis (4-, 3-, and 2-chamber) slices and three short axis (basal, mid-ventricular and apical) slices. FSENC strain imaging uses tagging gradients that are applied perpendicular to the long- and short axis slices, in a through-plane direction. The three short-axis cover16 myocardial segments and support segmental assessment of longitudinal strain (LS), while each of the three long-axis views (2‑, 3‑, and 4‑chamber) each cover 7 segments for circumferential strain (CS) analysis (Supplemental figure S1) [22]. Based on previous reports [11], we assessed fSENC as the percentage of segments with normal strain (% normal myocardium) using MyoStrain v5.1.5 (Myocardial Solutions, Inc., Morrisville, North Carolina), see Supplementary Methods for further details. % normal myocardium was calculated as the proportion of LV segments with normal segmental strain (defined as <  = -17), when considering both segments with calculated LS (normally 16 segments) and CS (normally 21 segments). % normal myocardium = ("number of segments with LS <  = -17" + "number of segments with CS <  = -17") / total number of analyzable LS + CS segments (normally 16 + 21 = 37). All fSENC analyses were performed by AWM.

Focal myocardial fibrosis was assessed on two-dimensional phase sensitive inversion recovery LGE images acquired in 10 mm short-axis slices covering the ventricles and three long-axis views starting 10 min after intravenous injection of 0.15 mmol/kg Clariscan (gadoterate meglumine). LGE was quantified using semiautomatic signal intensity thresholding set at 5 standard deviations (SD) above the remote myocardium. LGE presence, type (ischemic/nonischemic), volume (grams[g]) and American Heart Association (AHA) segment location were registered. Subendocardial or transmural LGE in a coronary-artery territory was categorized as an ischemic scar, whereas subepicardial or mid-wall LGE was categorized as a nonischemic scar. Small isolated LGE in the inferior insertion-point were considered benign and not categorized as focal scars [23]. Diffuse myocardial fibrosis was assessed by calculating the myocardial ECV from T1 maps acquired with Modified Look-Locker inversion recovery (MOLLI) sequences before and 15 min after the injection of intravenous contrast, and corrected for blood hematocrit using 5 s(3 s)3 s and 4 s(1 s)3 s(1 s)2 s sampling schemes, respectively [18]. Only the segments representing the midventricular septum were used for the calculation of ECV, and segments with artefacts or ischemic scars were excluded in accordance with SCMR consensus recommendations [6, 20]. The following formula was used to calculate ECV (%) from post- and pre-contrast maps:

$ECV=\left(100 \%-hematocrit(\%)\right)*\frac{\Delta \mathrm{R}1\mathrm{m}\mathrm{y}\mathrm{o}\mathrm{c}\mathrm{a}\mathrm{r}\mathrm{d}\mathrm{i}\mathrm{u}\mathrm{m}}{\Delta \mathrm{R}1\mathrm{b}\mathrm{l}\mathrm{o}\mathrm{o}\mathrm{d}}$, where Δ R1 myocardium = (1/post contrast T1 myocardium) – (1/ native T1 myocardium) and Δ R1 blood = (1/post contrast T1 blood) – (1/native T1 blood).

### Statistical analysis

Baseline characteristics of participants with and without focal fibrosis were compared and the data are reported as absolute numbers (percentages) or median (IQR). Continuous variables were compared using the Mann–Whitney U test and categorical variables using the Pearson Chi-Square test and the Fisher exact test, as appropriate. Spearman’s rank coefficient assessed correlations between the fibrosis metrics and metrics of systolic function and synchrony.

Linear and logistic regression analyses were used to examine relationships between both diffuse and focal fibrosis and strain metrics from CMR-FT. Absolute strain values were dichotomized at the sex-specific 25th percentile, and MD and ECV (%) at the sex-specific 75th percentile in the logistic regression analysis. Linear regression analyses were used to examine the relationship between GLS, GCS and fibrosis. Generalized linear models with an identity function and gaussian distribution were used to examine the relationship between fSENC % normal myocardium and both types of fibrosis. Robust standard deviation was used to account for potential heteroscedasticity. fSENC% normal myocardium was dichotomized at 80% in logistic regression analyses, a cutoff based on values reported as effective in prior studies [10, 11]. Generalized linear models with a log function and gamma distribution were used to examine the relationship between MD and both types of fibrosis. Nonlinear associations were assessed by constructing restricted cubic splines with 3 standard knots at the 10th, 50th and 90th study percentiles [24]. Sensitivity analyses were also performed using knots at ECV 25% and ECV 27% instead of the 50th percentile. The regression analyses were adjusted for a priori selected variables in four models; Model 1, unadjusted; Model 2, adjusted for age and sex; Model 3, adjusted for age, sex, body mass index (BMI), C-reactive protein (CRP), estimated glomerular filtration rate eGFR, hypertension, diabetes and smoking status; In Model 4, strain analyses were additionally adjusted for indexed LV end diastolic volume (LVEDVi) to account for potential effects of preload [25–27]. Likelihood Ratio Test was used to assess whether non-linear models provided a better fit than the linear model 3 and 4. Intraobserver analyses were performed by reanalysis of feature tracking and fSENC GLS and GCS in 20 randomly selected cases (see Supplemental Table S6).

## Results

### Baseline characteristics

The median age was 69.0 (IQR 68.6–69.3) years and 52% were male. Focal fibrosis was present in 45 (22.7%) of 198 participants with available LGE images. Nonischemic scars were detected in 27 participants and ischemic scars in 14 participants. Four had both scar types. Sixteen participants had focal scars in the septum, nine in the anterior wall, 29 in the lateral wall, 18 in the inferior wall and 19 in several regions. Five of the septal scars were ischemic.(Supplemental Table 3). The median size of the nonischemic scars was smaller than that of the ischemic scars (1.3 g (1.1–2.0) vs. 4.8 (2.0–10.0), p < 0.001). Participants with presence of LGE were more often male and had higher prevalence of hypertension (Table 1).

**Table 1:** Baseline characteristics according to presence of focal scars

	No focal scars	Focal scars present	p-value
	N = 153	N = 45	
Study site Akershus University Hospital, n (%)	99 (64.7)	32 (71.1)	0.42
Age, years	69.0 (68.6, 69.2)	69.0 (68.6, 69.3)	0.80
Male sex, n (%)	73 (47.7%)	30 (66.7%)	0.03
Body mass index, kg/m^2^	26.1 (23.5, 28.0)	27.1 (24.0, 29.4)	0.09
Current smoker, n (%)	8 (5.2%)	2 (4.4%)	0.83
Higher education, n (%)	85 (55.6)	20 (45.5)	0.24
Heart rate, beats/min	61 (56, 66)	60 (55, 66)	0.78
Systolic blood pressure, mmHg	134 (123, 148)	143 (130, 149)	0.048
Diastolic blood pressure, mmHg	74 (69, 81)	79 (74, 83)	0.008
Atrial fibrillation, n (%)*	10 (6.7)	7 (15.6)	0.06
Heart failure, n (%)	3 (2.0)	4 (8.9)	0.03
Diabetes mellitus, n (%)	7 (4.6%)	5 (11.1%)	0.11
Hypertension, n (%)	86 (56.2)	36 (80.0)	0.004
Medication			
Diuretics, n (%)	1 (0.7)	2 (4.4)	0.067
β blockers, n (%)	5 (3.3)	5 (11.1)	0.04
Calcium antagonists, n (%)	6 (3.9)	2 (4.4)	0.88
ACE-I/ARB, n (%)	24 (15.7)	7 (15.6)	0.98
Other antihypertensives, n (%)	0 (0.0%)	1 (2.2%)	0.07
Statins, n (%)	24 (15.7)	7 (15.6)	0.98
Laboratory measurements			
CRP ≥ 3 mg/L, n (%)	22 (14.5%)	10 (22.2)	0.29
HbA1c, %	37.4 (35.3, 40.0)	38.0 (35.9, 40.6)	0.53
Glucose, mg/dL^b^	5.5 (5.0, 5.9)	5.7 (5.2, 6.0)	0.22
White blood count, 10^9^/L	5.2 (4.4, 6.0)	5.8 (4.7, 7.2)	0.004
Hemoglobin, g/dL	14.0 (13.3, 14.9)	14.5 (13.8, 15.3)	0.02
Total cholesterol, mg/dL**	5.4 (4.8, 6.0)	5.2 (4.5, 6.0)	0.25
HDL cholesterol, mg/dL**	1.6 (1.3, 2.0)	1.5 (1.2, 2.0)	0.19
Triglycerides, mg/dL	1.2 (0.9, 1.6)	1.3 (0.8, 1.6)	0.93
eGFR, mL/min/1.73m^2^	93.2 (85.2, 95.7)	90.3 (82.5, 93.5)	0.07

ACEi, angiotensin-converting enzyme inhibitor; ARB, angiotensin II receptor blocker; CRP, C-reactive protein; eGFR, estimated glomerular filtration rate; HbA_1c_, glycosylated hemoglobin; HDL, high-density lipoprotein

^*^History of atrial fibrillation reported prior to CMR

^**^To convert glucose concentrations from mg/dL to mmol/L, multiply by 0.05556. To convert triglyceride concentrations from mg/dL to mmol/L, multiply by 0.01129. To convert cholesterol concentrations from mg/dL to mmol/L, multiply by 0.02586

In the 192 participants with adequate T1 maps, the median ECV was 26.7% (25.4, 28.3) for females and 25.3 (23.7, 26.9) for males. Median GLS was -17.3% (-18.7, -15.8), median GCS was -17.7% (-19.2, -16.0) and median MD was 80.4 ms (61.8,104.6) among the 185 participants with SSFP images of quality sufficient for feature tracking. Of 189 participants with available and adequate SENC images, all 37 segments were analyzable in 186 participants. Two participants had 31 analyzable segments, and one participant had 30 analyzable segments. Reason for exclusion of segments was lack of segment coverage.

fSENC % normal myocardium above 80% was present in 42 (22.2%) participants with available fSENC images. GLS and GCS were normally distributed (Supplemental Figures S1 and S2), whereas fSENC % normal myocardium exhibited a right-skewed and MD a left-skewed distribution (Supplemental Figs. 3 and 4).

Participants with focal scars had worse median GLS (-16.1% (-18.2, -14.8) vs. -17.5% (-19.1, -16–1), p = 0.002) and GCS (-16.3% (-18.8, -15.1) vs. -18% (-19.4, -16.4), p = 0.006) compared to those without focal fibrosis. The median MD was significantly higher among participants with focal fibrosis (89.0 ms (71.2, 121.1) vs (78.0 ms (60.4, 97.3), p = 0.008). The five outliers with the highest MD in the group without scars did not have bundle branch block.

**Fig. 2:**
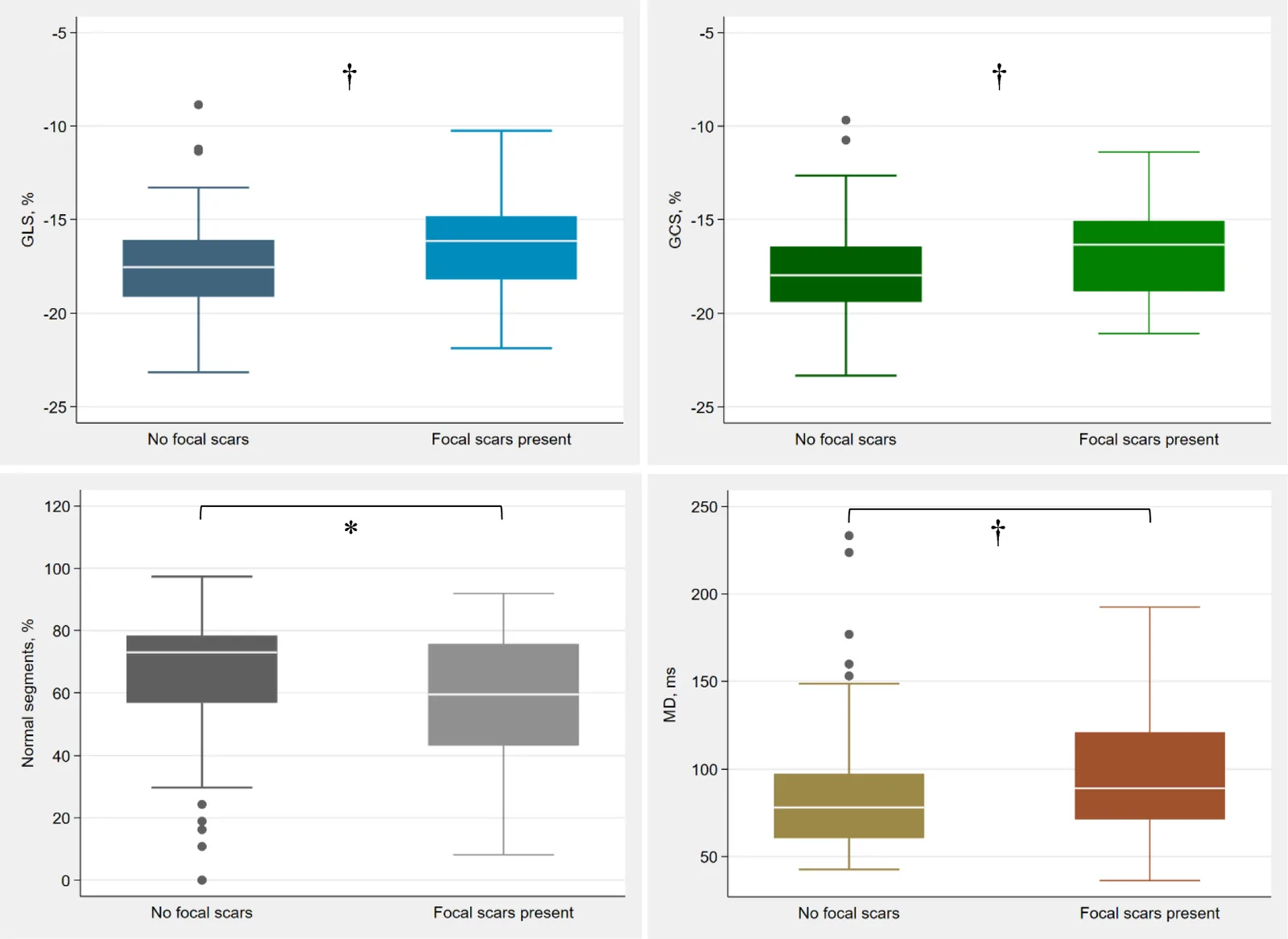
Boxplot of global longitudinal strain, global circumferential strain, mechanical dispersion and % normal segments assessed by fSENC stratified by presence of focal myocardial scar. Groups were compared using the two-sided Mann–Whitney *U* test. Significance level is denoted as *P < 0.05, †*P* < 0.01, and ‡*P* < 0.001. GLS, global longitudinal strain; GCS, global circumferential strain; MD, mechanical dispersion

Participants with focal fibrosis had significantly lower median fSENC % normal myocardium compared to those with no detectable LGE (59.5% (43.2–75.7) vs. 73.0 (56.8–78.4), p = 0.01) (Table 2 and Fig. 2).

**Table 2:** CMR Characteristics According to Presence of Myocardial Scar

	All with adequate LGE-images		No Focal scar	Focal scar present	P-value
	N	198	153	45	
Scar volume, g	198	0.0 (0.0, 0.0)	-	1.7 (1.1, 4.0)	NA
Septal ECV, %	192	26.0 (24.4, 27.7)	26.2 (24.7, 27.6)	25.9 (24.0, 28.4)	0.77
LV mass indexed, g/m^2^	199	46.6 (40.9, 55.1)	45.0 (40.6, 51.5)	56.4 (48.8, 64.4)	0.001
LV ejection fraction, %	198	59. 9 (55.0, 64.1)	60.1 (56.4, 63.8)	56.7 (51.0, 64.4)	0.054
LVGLS, %	185	-17.3 (-18.7, -15.8)	-17.5 (-19.1, -16.1)	-16.1 (-18.2, -14.8)	0.002
LVGCS, %	185	-17.7 (-19.2, -16.0)	-18.0 (-19.4, -16.4)	-16.3 (-18.8, -15.1)	0.006
% normal segments, %	189	70.3 (51.4, 78.4)	73.0 (56.8–78.4)	59.5 (43.2–75.7)	0.01
n (%) with % normal segments below cut-off*	189	41.0 (21.9)	34.0 (23.6)	7.0 (16.7)	0.35
MD, ms	185	80.4 (61.8,104.6)	78.0 (60.4, 97.3)	89.0 (71.2, 121.1)	0.008

Values are medians with interquartile intervals

CMR, cardiovascular magnetic resonance; ECV, extracellular volume; LGE, late gadolinium enhancement; LV, left ventricle; LVGCS, left ventricular circumferential strain; LVGLS, left ventricular longitudinal strain; MD, mechanical dispersion

^*^Participants with more than 80% of segments with strain from fast strain-encoded images ≤ -17%

### Correlation between CMR indices of fibrosis and strain and mechanical dispersion

Presence of focal fibrosis correlated with worse GLS (rho = 0.23, p = 0.002), worse GCS (rho = 0.20, p = 0.001), and lower fSENC % normal myocardium (rho -0.20, p = 0.001). There was no significant correlation between septal ECV and GLS (rho = -0.11, p = 0.16) or GCS (rho = -0.10, p = 0.20), but septal ECV correlated positively with fSENC % normal myocardium (rho = 0.22, p = 0.002). Presence of focal scars (rho = 0.20, p = 0.007) but not ECV (rho = 0.07, p = 0.36) correlated with MD.

### Association between CMR indices of fibrosis and strain from CMR-FT

**Fig. 3:**
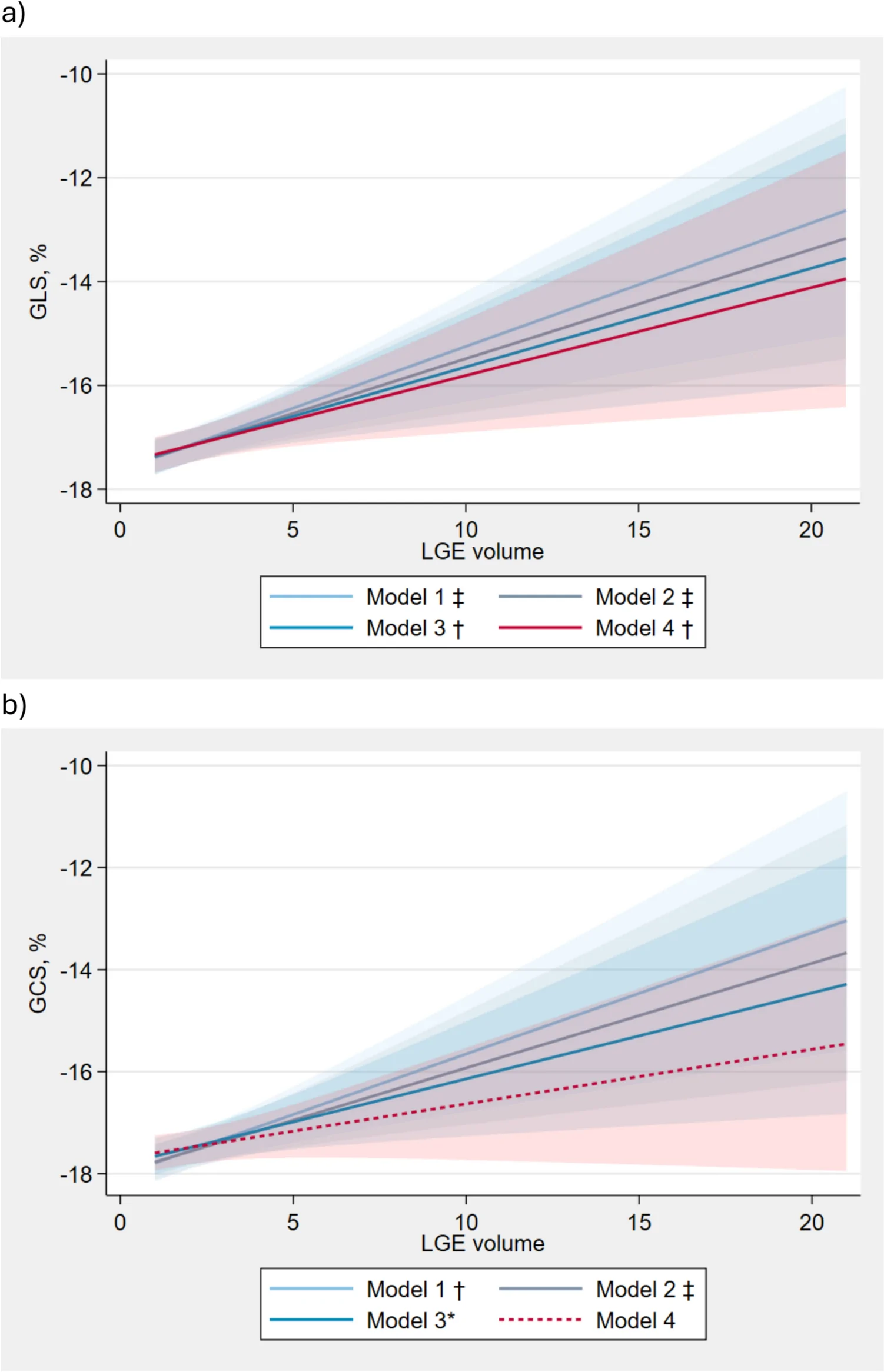
Linear association between LGE volume (g) and a) GLS (%) and b) GCS (%) across four different models with 95% confidence intervals. ^*^P < 0.05, ^†^*P* < 0.01, ^‡^*P* < 0.001. Model 1, unadjusted; Model 2, adjusted for age and sex; Model 3, adjusted for age, sex, BMI, CRP, eGFR, hypertension, diabetes and smoking status; Model 4, adjusted for model 3 and LVEDVi

Both presence and volume of LGE measured in gram were associated with worse GLS and GCS. For GLS, the association remained in all models, but for GCS the association was attenuated and not significant when adjusting for LVEDi in the fourth model expansion (Table 3 and Fig. 3).

**Table 3:** Association of LGE presence/volume and ECV with strain^a^

Beta coefficients from linear regression analyses					P-value for nonlinearity^b^
	Model 1 B (95% CI)	Model 2 B (95% CI)	Model 3 B (95% CI)	Model 4 B (95% CI)	
GLS					
LGE presence	1.29 (0.50, 2.08) ^†^	1.08 (0.31, 1.85) ^†^	0.96 (0.17, 1.75)^*^	0.83 (0.02, 1.63)^*^	Not tested^c^
LGE, gram	0.24 (0.11, 0.36) ^‡^	0.21 (0.09, 0.33) †^‡^	0.19 (0.07, 0.32)^†^	0.17 (0.04, 0.30) ^*^	Not tested^c^
ECV	- 0.07 (-0.22, 0.09)	0.043 (-0.11, 0.20)	0.07 (-0.09, 0,24)	0.05 (-0.11, 0.21)	0.001
GCS					
LGE presence	1.28 (0.45, 2.12) ^†^	1.08 (0.26, 1.90) ^*^	1.01 (0.18, 1.84)^*^	0.66 (-0.15, 1.45)	Not tested^c^
LGE, gram	0.24 (0.11, 0.37) ^‡^	0.20 (0.08, 0.33) ^†^	0.17 (0.04, 0.30)^*^	0.11 (-0.02, 0.24)	Not tested^c^
ECV	-0.04 (-0.2, 0.12)	0.05 (-0.11, 0.22)	0.10 (-0.06, 0.27)	0.03 (-0.13, 0.20)	0.02

B, beta coefficient; CI, confidence interval; ECV, extracellular volume; LGE, late gadolinium enhancement; GCS, left ventricular circumferential strain; GLS, left ventricular longitudinal strain

^*^P < 0.05, ^†^*P* < 0.01, ^‡^*P* < 0.001

^a^ strain values were not converted to absolute values

^b^P-value for comparison of fully adjusted linear regression analysis with spline regression analysis with 3 knots with Likelihood Ratio Test

^c^Spline analyses were not performed due to an insufficient number of participants with detectable LGE

Model 1, unadjusted; Model 2, adjusted for age and sex; Model 3, adjusted for age, sex, BMI, CRP, eGFR, hypertension, diabetes and smoking status; Model 4, adjusted for model 3 and LVEDVi

**Fig. 4:**
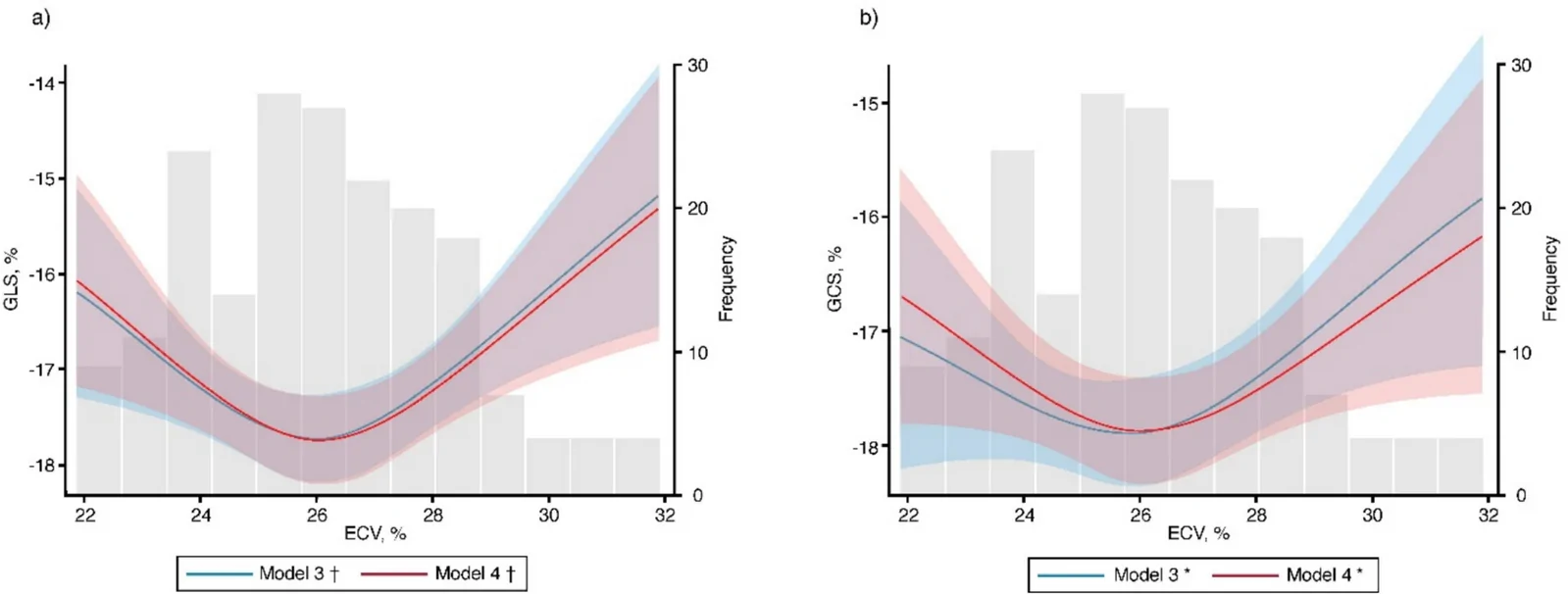
Nonlinear association between ECV and GLS to the left and GCS to the left across two different models with 95% confidence intervals. ^*^P < 0.05, ^†^*P* < 0.01, ^‡^*P* < 0.001. Model 3, adjusted for age, sex, BMI, CRP, eGFR, hypertension, diabetes and smoking status; Model 4, adjusted for model 3 and LVEDVi. Frequency of ECV values illustrated with a histogram. ECV, extracellular volume; GCS, global circumferential strain; GLS, global longitudinal strain

Nonlinear models were superior to linear models for describing the relationship between ECV and both GLS (p for nonlinearity = 0.001 for both model 3 and 4) and GCS (p for nonlinearity = 0.04 for model 3 and 0.02 for model 4). Both strain parameters exhibited a U-shaped nonlinear association with ECV, with worse strain at ECV values above and below ~ 26% (Table 3) (Fig. 4). Sensitivity analysis using knots at the 10th and 90th percentiles and substituting the 50th percentile with 25% and 27% in separate models, also demonstrated a u-shaped nonlinear associations that were superior to the linear models (p for nonlinearity < 0.05) (Supplemental Figure S6 and S7).

Presence and volume of LGE were associated with GLS and GCS values in the sex-specific worst quartile (i.e. least negative). For GLS, the association remained significant in all models, but the association between LGE volume and GCS was attenuated and no longer significant when adjusting for cardiovascular risk factors. There was no association between ECV and strain values within the sex-specific worst quartile (i.e. least negative; Supplemental Table 1S).

### Association between CMR indices of fibrosis and strain assessed by fSENC

**Fig. 5:**
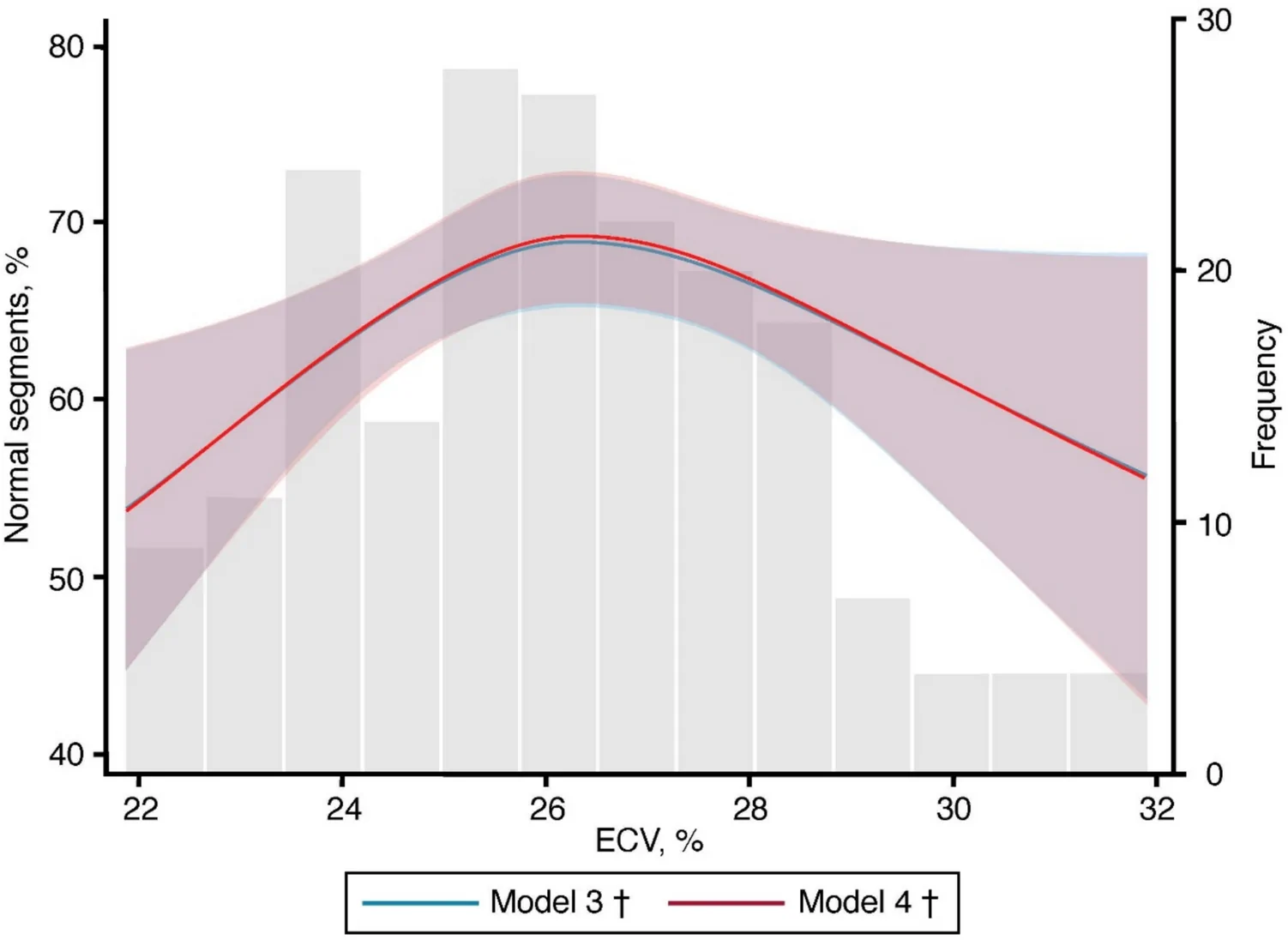
Nonlinear association between ECV and fSENC % normal myocardium across two different models with 95% confidence intervals. ^*^P < 0.05, ^†^*P* < 0.01, ^‡^*P* < 0.001. Model 3, adjusted for age, sex, BMI, CRP, eGFR, hypertension, diabetes and smoking status; Model 4, adjusted for model 3 and LVEDVi. ECV, extracellular volume

Presence and volume of focal scars were associated with lower fSENC % normal myocardium in all linear regression models (Table 4), apart from an attenuated association for scar presence in the model adjusting for common CV risk factors. The unadjusted association between ECV and fSENC % normal myocardium was attenuated in all other models (Table 4). As with GLS, fSENC % normal myocardium displayed a non-linear relationship with ECV with an inflection point at ECV of ~ 26% (Fig. 5) and the nonlinear model was superior to linear model for describing the relationship (p for nonlinearity = 0.005 for model 3 and 0.006 for model 4). In logistic regression analyses, neither ECV, LGE presence nor volume were associated with fSENC % normal myocardium < 80% (Supplemental Table S2).

**Table 4:** Association of LGE presence/volume and ECV with fSENC % normal segments

Beta coefficients from linear regression analyses					P-value for nonlinearity^a^
	B (95% CI)	Model 2 B (95% CI)	Model 3 B (95% CI)	Model 4 B (95% CI)	
fSENC % normal segments					
LGE presence	-8.05 (-14.85, -1.26)*	-6.79 (-13.58, -0.01)	-6.05 (-12.44, 0.33)	-6.68 (-13.35, -0.04)*	Not tested^b^
LGE, gram	-2.18 (-3.71, -0.65)^†^	-1.85 (-3.45, -0.25)*	-1.49 (-2.82, -0.17)*	-1.66 (-3.14, -0.18)*	Not tested^b^
ECV	1.65 (0.22, 3.09)*	1.09 (-0.31, 2.51)	0.59 (-0.77, 1.97)	0.55 (-0.85, 1.97)	0.006^†^

B, beta coefficient; CI, confidence interval; ECV, extracellular volume; fSENC, fast strain-encoded, LGE, late gadolinium enhancement

^*^P < 0.05, ^†^*P* < 0.01, ^‡^*P* < 0.001

^a^P-value for comparison of fully adjusted linear regression analysis with spline regression analysis with 3 knots with Likelihood Ratio Test

^b^Spline analyses were not performed due to an insufficient number of participants with detectable LGE

Model 1, unadjusted; Model 2, adjusted for age and sex; Model 3, adjusted for age, sex, BMI, CRP, eGFR, hypertension, diabetes and smoking status. Model 4 adjusted for model 3 and LVEDVi

### Association between CMR indices of fibrosis and MD

In the linear regression analysis, the presence of LGE was associated with higher MD in the unadjusted model and the model adjusted for age and sex, but the association was attenuated after further adjustment for common CV risk factors. ECV was not associated with higher MD (Supplemental Table S3). When MD was dichotomized at the sex-specific upper quartile, presence and volume of LGE were associated with MD in all models (Table 5).

**Table 5:** Association of LGE presence/volume and ECV with MD

Odds ratio from logistic regression analyses				P-value for nonlinearity^a^
	Model 1 OR (95% CI)	Model 2 OR (95% CI)	Model 3 OR (95% CI)	
MD^c^				
LGE presence	2.53 (1.20, 5.36)*	2.54 (1.18, 5.46)*	2.64 (1.18, 5.94)*	Not tested^b^
LGE, gram	1.19 (1.03, 1.37) *	1.18 (1.03, 1.35) *	1.22 (1.05, 1.42)*	Not tested^b^
ECV	1.11 (0.95–1.30)	1.13 (0.96–1.33)	1.13 (0.95, 1.34)	0.21

CI, confidence interval; ECV, extracellular volume LGE, late gadolinium enhancement, MD, mechanical dispersion; OR, odds ratio

^*^P < 0.05, ^†^*P* < 0.01, ^‡^*P* < 0.001

^a^P-value for comparison of fully adjusted linear regression analysis with spline regression analysis with 3 knots with Likelihood Ratio Test

^b^Spline analyses were not performed due to an insufficient number of participants with detectable LGE

^c^ MD dichotomized at the sex-specific upper quartile (93.4 ms for women and 111.5 for men)

Model 1, unadjusted; Model 2, adjusted for age and sex; Model 3, adjusted for age, sex, BMI, CRP, eGFR, hypertension, diabetes and smoking status

## Discussion

In this CMR study of elderly participants from the general population, we demonstrate the following key findings: 1) focal myocardial scars are associated with worse GLS and GCS assessed by CMR-FT as well as a lower % normal myocardium assessed by fSENC, 2) focal scars are associated with worse myocardial synchrony as assessed by MD, and 3) diffuse fibrosis displays a nonlinear relationship with both global longitudinal and circumferential deformation and fSENC % normal myocardium.

### Association between focal myocardial fibrosis and myocardial function

Myocardial scar tissue is associated with morbidity and mortality. Although scar tissue is non-contractile, studies in the general population show that individuals with unrecognized myocardial scars often have EF within the normal range despite between-group differences [28]. Myocardial strain is a more sensitive measure of myocardial function [25]. Associations between focal scars and echocardiographically assessed strain are documented in disease cohorts [14, 29–31], but corresponding data from healthier community samples are limited. The participants in this study had no known coronary disease 4–7 years prior to the CMR examination, still, focal ischemic or non-ischemic scars were present in 23% at the time of CMR. Even in this relatively healthy, elderly cohort, both the presence and volume of LGE were associated with worse GLS assessed by CMR-FT and lower % normal myocardium assessed by fSENC, indicating that subclinical focal scars may affect the myocardial contractile apparatus, causing measurable changes in the LV systolic function.

#### GLS and GCS by CMR-feature tracking

In our study, both the presence of focal scars and scar volume were associated with worse GLS and GCS by CMR-FT independently of common cardiovascular risk factors. Previous studies have shown that GLS and GCS are load dependent [32]. In our cohort, the association between scars and GLS persisted also upon additional adjustment for LVEDVi, while the addition attenuated the association with GCS. The role of LVEDVi in the fibrosis-strain association is likely complex: higher LVEDVi may, for instance, (i) reflect favorable athletic remodelling with both lower ECV and lower resting strain (confounder) and can also (ii) reflect pathological changes where, where sustained elevations in wall stress lead to pathological remodeling with higher ECV that in turn alters LV geometry and myocardial mechanics (mediator). As the current population is selected from a general, older population, with a relatively high prevalence of hypertension, the mediating role of LVEDVi is likely a substantial driver.Adjustment for LVEDVi could therefore partly block the pathway between fibrosis and mechanics. Interpretations of LVEDVi adjusted results should therefore remain cautious.

#### fSENC

fSENC % normal myocardium reflects both longitudinal and circumferential segmental strain measurements, and may provide a more comprehensive assessment of the complex three-dimensional systolic function compared to isolated global measurements like GLS and GCS. Studies indicate that fSENC % normal myocardium may be a promising predictor of heart failure and cardiotoxicity [10, 11], but few studies have assessed the association between this marker and myocardial fibrosis. In a study of patients with left ventricular hypertrophy, fSENC % normal myocardium demonstrated discriminative value between athletes and both individuals with hypertrophic and hypertensive cardiomyopathy, and a moderate correlation with non-ischemic scars [33]. Extending these observations to an elderly general population, we found a linear association between fSENC % normal myocardium and presence and volume of focal scars. Previous studies have indicated that a cut-off value of < 80% normal myocardium is optimal for detection and prediction of heart failure and cardiotoxicity [10, 11]. In our study, dichotomizing fSENC % normal myocardium at 80% obscured the linear association, and this cut-off value may be less sensitive in capturing subtle relationships between focal scarring and myocardial function.

### Association between focal fibrosis and mechanical dispersion

Echocardiographic studies have shown that increased MD predicts ventricular arrythmias and sudden cardiac death in several cardiac diseases [9]. Myocardial dyssynchrony may occur because of conduction disturbances or myocardial scarring, both of which are known substrates for cardiac arrythmias [9, 14]. As focal scars affect the myocardium regionally, there is reason to believe that this causes regional differences in contraction, leading to dyssynchronous contraction. Echocardiographic studies have demonstrated that MD is correlated with focal scar burden in ischemic and hypertrophic cardiomyopathy [14, 34], whereas a study of patients with non-ischemic cardiomyopathies found no relationship between MD and myocardial scars [35]. By assessing MD with echocardiography and CMR, our group has previously demonstrated an association between MD and both cardiovascular risk factors and biomarkers of myocardial injury in the general population [21, 36], but so far, no studies have assessed the association between focal scars and dyssynchrony in the general population. In the current study, participants with focal scars had significantly higher MD compared to those without. Although the association between increasing MD and focal scars was attenuated when adjusting for common cardiovascular risk factors, MD above the sex specific upper quartile was associated with focal scarring, implying a threshold value in a subclinical setting. However, the value for dichotomization in our cohort is data-driven as there are no established clinical cut-off values for MD. MD values reported by echocardiographic and CMR studies indicate that the values from the different modalities are not interchangeable [21], and larger studies and long-term follow up will be necessary to assess optimal cut-off values and clinical importance of higher MD in the general population.

### Association between diffuse myocardial fibrosis and myocardial function

In many chronic myocardial and systemic diseases, as well as in aging, diffuse cardiac fibrosis contributes to increased myocardial stiffness, distorted architecture and impaired systolic and diastolic function [1]. ECV assessed by CMR is a marker of interstitial fibrosis that correlates strongly with histopathology [37] and has incremental prognostic value to LGE in myocardial disease [38]. Subclinical diffuse myocardial fibrosis and change in myocardial function may precede clinical symptoms, but few studies have assessed the relationship between diffuse fibrosis and strain in the general population. In the Multi-Ethnic Study of Atherosclerosis (MESA) study, a linear association between diffuse fibrosis assessed by postcontrast T1 times and worse GCS was demonstrated [39]. However, ECV, a more robust marker of diffuse fibrosis [37], could not be estimated as hematocrit values were unavailable. Two recent studies of clinically referred patients assessed the relationship between diffuse myocardial fibrosis and strain assessed by feature tracking. Frøjd et al. demonstrated that both ECV and GLS were associated with adverse CV outcomes but were only weakly correlated [40], whereas Jin et al. found that both GLS and GCS associated with ECV values exceeding 2SD of normal controls [41].

Corresponding to the findings of Jin et al., we found a non-linear association between ECV and myocardial deformation, where GLS, GCS and fSENC % normal myocardium in all models demonstrated an inflection point at 26%. A systematic review and meta-analysis of healthy subjects from 2019 report a pooled ECV estimate of 26% with a wide normal range (20–32%), noting an overlap with ECV values reported in myocardial diseases [42]. The restricted ECV range in the currently studied population likely contributed to the lack of a linear association between ECV and strain in this cohort. Such associations may become more apparent in cohorts with a wider range of ECV values, including clearly elevated ECV values. Interestingly, the threshold value in our cohort fell well within what is considered normal range. The upper arm of this curve supports that diffuse fibrosis may affect strain beyond a certain degree of diffuse extracellular expansion. Paradoxically, lower ECV values were also associated with poor strain, and this is more difficult to interpret biologically. One potential explanation could be that individuals with higher LV volumes might rely less on myocardial contraction at rest, but this U-shaped non-linear association persisted even after adjusting for LVEDVi. Of note, in 2023 Thavendiranathan, et al. found a similar U-shaped relationship between ECV and future Cancer Therapy–Related Cardiac Dysfunction in breast cancer patients [43].

Histological validation of CMR-derived ECV as marker of diffuse fibrosis has largely been performed in cohorts with established cardiac disease [44–46] with comparatively limited validation in the lower ECV range typical for population-based samples. This constrains inference about what low ECV represents biologically and warrants cautious interpretation of associations between lower ECV values and strain.

Diffuse fibrosis is, in contrast to focal scars, more homogenously spread throughout the myocardium without regional predilection. Therefore, myocardial contractility may be expected to be affected in approximately equal measures across all myocardial segments, consistent with our findings of no significant association between ECV and MD in both unadjusted and fully adjusted models.

### Clinical implications

Both feature tracking and fSENC provide valuable approaches to assessment of myocardial function, each with specific advantages: Whereas strain can be derived by feature tracking from routinely acquired standard cine sequences, fSENC requires additional scans but provides rapid image acquisition and analysis of function, and the choice between them must be tailored to the clinical scenario. Our findings support that myocardial fibrosis is associated with a preclinical stage of cardiovascular disease, thereby contributing to improved pathophysiological understanding of disease progression. However, longitudinal studies that link these findings to clinical endpoints, as well as validation in other cohorts, are required before CMR can be considered for use as a screening tool or risk-stratification tool in the elderly.

## Limitations

Causal relationships between fibrosis and cardiac function cannot be inferred with certainty due to the cross-sectional nature of the study. MD assessed by CMR and fSENC are emerging imaging biomarkers, and the findings of our study need to be validated in other cohorts. The limited number of participants with focal scars or elevated ECV levels may have reduced the statistical power of the analyses. Still, this pattern may reflect the typical distribution within an elderly general population. Even though septal ECV is a commonly used and well validated measure of global diffuse myocardial fibrosis, we acknowledge that using a septal measure as a representative of the entire myocardium may fail to capture regional variations in diffuse fibrosis distribution. Participants were without known coronary artery disease at 5–7 years before CMR, interim CVD was not accounted for in the analyses, and the prevalence of focal scars might not only represent asymptomatic disease. Although the exact timing of focal scar development is uncertain, we believe that the exclusion of individuals with known CAD at baseline ensures a general population cohort, and that interim disease represents natural occurrence of disease in this population. Focal scars in this cohort encompasses both ischemic and non-ischemic scar types, which differ in pathophysiology and significance. Combining these scar types into a heterogenic group may limit mechanistic interpretation. Our cohort consists of older participants with Caucasian origin, and the findings are less generalizable to a multiethnic general population cohort covering a wider age range. Further, diastolic function was not measured. Finally, the long-term implications of our findings remain to be established, however long-term follow-up of the ACE study cohort is ongoing.

## Conclusion

In an elderly general population, focal myocardial fibrosis is associated with worse LV strain assessed by CMR-FT, lower fSENC percentage of normally deforming myocardium, and worse synchrony assessed by MD. There was a non-linear association between diffuse fibrosis and strain with worse cardiac deformation beyond a nadir of ~ 26%. These findings demonstrate that both focal and diffuse myocardial fibrosis are associated with subclinical myocardial dysfunction, highlighting the potential for early detection and risk stratification of CVD.

## Supplementary Information

Below is the link to the electronic supplementary material.

Supplementary file 1.

## Data Availability

Data used is internal to Kristian Gerard Jebsen Center for Cardiac Biomarkers.

## Abbreviations

CMR: Cardiovascular magnetic resonance
LGE: Late gadolinium enhancement
ECV: Extracellular volume fraction
MD: Mechanical dispersion
LV: Left Ventricular
CMR-FT: CMR with feature-tracking
fSENC: Fast strain-ENCoded imaging
ACE: Akershus cardiac examination
eGFR: Estimated glomerular filtratrion rate
CAD: Coronary artery disease
SCMR: Society of cardiovascular magnetic resonance
SSFP: Steady-steate free precession
GLS: Global longitudinal strain
GCS: Global circumferential strain
AHA: American heart association
EF: Ejection fraction
MOLLI: Modified look-locker inversion recovery
IQR: Interquartile range
BMI: Body mass index
CRP: C-reactive protein
LVEDVi: Left ventricular end-diastolic volume indexed
MESA: Multi-ethnic study of atherosclerosis

## Declarations

### Clinical trials

Gov ID NCT01555411, prospective.

## References

[1] López B, Ravassa S, Moreno MU, José GS, Beaumont J, González A et al (2021) Diffuse myocardial fibrosis: mechanisms, diagnosis and therapeutic approaches. Nat Rev Cardiol 18(7):479–498 33568808 10.1038/s41569-020-00504-1

[2] Shanbhag SM, Aspelund T, Greve AM, Thorgeirsson G, Schelbert EB, Cao JJ et al (2016) Prevalence and prognosis of non-ischemic patterns of late gadolinium enhancement in older adults by cardiovascular MR in the ICELAND-MI study. J Cardiovasc Magn Reson 18(1):1–226732096

[3] Seliger SL, Hong SN, Christenson RH, Kronmal R, Daniels LB, Lima JAC et al (2017) High-sensitive cardiac troponin T as an early biochemical signature for clinical and subclinical heart failure: MESA (multi-ethnic study of atherosclerosis). Circulation 135(16):1494–1505 28159799 10.1161/CIRCULATIONAHA.116.025505 PMC5401621

[4] Marques MD, Weinberg R, Kapoor S, Ostovaneh MR, Kato Y, Liu CY et al (2022) Myocardial fibrosis by T1 mapping magnetic resonance imaging predicts incident cardiovascular events and all-cause mortality: the multi-ethnic study of atherosclerosis. Eur Heart J Cardiovasc Imaging 23(10):1407–1416 35147665 10.1093/ehjci/jeac010 PMC9463991

[5] Espeland T, Lunde IG, B HA, Gullestad L, Aakhus S (2018) Myocardial fibrosis. Tidsskr Nor Laegeforen. 138(16). 10.4045/tidsskr.17.1027 30344312

[6] Puntmann VO, Valbuena S, Hinojar R, Petersen SE, Greenwood JP, Kramer CM et al (2018) Society for cardiovascular magnetic resonance (SCMR) expert consensus for CMR imaging endpoints in clinical research: part I - analytical validation and clinical qualification. J Cardiovasc Magn Reson 20(1):67 30231886 10.1186/s12968-018-0484-5 PMC6147157

[7] Hundley WG, Bluemke DA, Bogaert J, Flamm SD, Fontana M, Friedrich MG et al (2022) Society for cardiovascular magnetic resonance (SCMR) guidelines for reporting cardiovascular magnetic resonance examinations. J Cardiovasc Magn Reson 24(1):29 35484555 10.1186/s12968-021-00827-z PMC9052489

[8] Singh A, Voss WB, Lentz RW, Thomas JD, Akhter N (2019) The diagnostic and prognostic value of echocardiographic strain. JAMA Cardiol 4(6):580–588 31042262 10.1001/jamacardio.2019.1152

[9] Kawakami H, Nerlekar N, Haugaa KH, Edvardsen T, Marwick TH (2020) Prediction of ventricular arrhythmias with left ventricular mechanical dispersion: a systematic review and meta-analysis. JACC Cardiovasc Imaging 13(2, Part 2):562–572 31202762 10.1016/j.jcmg.2019.03.025

[10] Giusca S, Korosoglou G, Montenbruck M, Gersak B, Schwarz AK, Esch S et al (2021) Multiparametric early detection and prediction of cardiotoxicity using myocardial strain, T1 and T2 mapping, and biochemical markers a longitudinal cardiac resonance imaging study during 2 years of follow-up. Circ-Cardiovasc Imag 14(6):473–484 10.1161/CIRCIMAGING.121.012459 PMC8208092 34126756

[11] Korosoglou G, Giusca S, Montenbruck M, Patel AR, Lapinskas T, Gotze C et al (2021) Fast strain-encoded cardiac magnetic resonance for diagnostic classification and risk stratification of heart failure patients. JACC Cardiovasc Imaging 14(6):1177–1188 33454266 10.1016/j.jcmg.2020.10.024

[12] Bucius P, Erley J, Tanacli R, Zieschang V, Giusca S, Korosoglou G et al (2020) Comparison of feature tracking, fast-SENC, and myocardial tagging for global and segmental left ventricular strain. ESC Heart Fail 7(2):523–532 31800152 10.1002/ehf2.12576 PMC7160507

[13] Zhao X, Zhang L, Wang L, Zhang W, Song Y, Zhao X et al (2025) Magnetic resonance imaging quantification of left ventricular mechanical dispersion and scar heterogeneity optimize risk stratification after myocardial infarction. BMC Cardiovasc Disord 25(1):2 39755625 10.1186/s12872-024-04451-4 PMC11699822

[14] Håland TF, Almaas VM, Hasselberg NE, Saberniak J, Leren IS, Hopp E et al (2016) Strain echocardiography is related to fibrosis and ventricular arrhythmias in hypertrophic cardiomyopathy. Eur Heart J Cardiovasc Imaging 17:613–621 26873460 10.1093/ehjci/jew005 PMC4871235

[15] Gotschy A, Jordan S, Stoeck CT, von Deuster C, Peer T, Gastl M et al (2023) Diffuse myocardial fibrosis precedes subclinical functional myocardial impairment and provides prognostic information in systemic sclerosis. Eur Heart J Cardiovasc Imaging 24(3):373–382 35639682 10.1093/ehjci/jeac094

[16] Fischer K, Obrist SJ, Erne SA, Stark AW, Marggraf M, Kaneko K et al (2020) Feature tracking myocardial strain incrementally improves prognostication in myocarditis beyond traditional CMR imaging features. JACC Cardiovasc Imaging 13(9):1891–1901 32682718 10.1016/j.jcmg.2020.04.025

[17] Kammerlander AA, Donà C, Nitsche C, Koschutnik M, Schönbauer R, Duca F et al (2020) Feature tracking of global longitudinal strain by using cardiovascular MRI improves risk stratification in heart failure with preserved ejection fraction. Radiology 296(2):290–298 32484413 10.1148/radiol.2020200195

[18] Wimalanathan T, Paus MF, Brox Skranes J, Berge T, Tveit A, Rosjo H, et al. (2024) Associations between growth differentiation factor 15, cardiac troponin T, and N-terminal pro-B-type natriuretic peptide, and future myocardial fibrosis assessed by cardiac magnetic resonance imaging: data from the akershus cardiac examination 1950 Study. J Appl Lab Med. 10.1093/jalm/jfae145 39707823

[19] Berge T, Vigen T, Pervez MO, Ihle-Hansen H, Lyngbakken MN, Omland T et al (2015) Heart and brain interactions-the Akershus Cardiac Examination (ACE) 1950 study design. Scand Cardiovasc J 49(6):308–315 26364744 10.3109/14017431.2015.1086813

[20] Messroghli DR, Moon JC, Ferreira VM, Grosse-Wortmann L, He T, Kellman P et al (2017) Clinical recommendations for cardiovascular magnetic resonance mapping of T1, T2, T2* and extracellular volume: a consensus statement by the society for cardiovascular magnetic resonance (SCMR) endorsed by the European Association for Cardiovascular Imaging (EACVI). J Cardiovasc Magn Reson 19(1):75 28992817 10.1186/s12968-017-0389-8 PMC5633041

[21] Sulkowska J, Melles AW, Skranes JB, Berge T, Tveit A, Rosjo H, et al. (2024) Cardiac troponin T associates with left ventricular function and synchrony assessed by CMR in the general population: results from the Akershus Cardiac Examination 1950 study. Eur Heart J Imaging Methods Pract.2(3):qyae078. 10.1093/ehjimp/qyae078 PMC11441316 39351316

[22] Riffel JH, Siry D, Salatzki J, Andre F, Ochs M, Weberling LD et al (2021) Feasibility of fast cardiovascular magnetic resonance strain imaging in patients presenting with acute chest pain. PLoS ONE 16(5):e0251040 33939756 10.1371/journal.pone.0251040 PMC8092784

[23] Grigoratos C, Pantano A, Meschisi M, Gaeta R, Ait-Ali L, Barison A et al (2020) Clinical importance of late gadolinium enhancement at right ventricular insertion points in otherwise normal hearts. Int J Cardiovasc Imaging 36(5):913–920 32026265 10.1007/s10554-020-01783-y

[24] Harrell F. Regression modeling strategies: with applications to linear models, logistic regression, and survival analysis2010.

[25] Otto AS, Oliver R, Marta C, Ladislav V, Espen WR, Jens-Uwe V (2025) Myocardial strain imaging: theory, current practice, and the future. JACC Cardiovasc Imaging 18(3):340–381 39269417 10.1016/j.jcmg.2024.07.011

[26] Ahn HS, Kim YK, Song HC, Choi EJ, Kim GH, Cho JS et al (2017) The impact of preload on 3-dimensional deformation parameters: principal strain, twist and torsion. Cardiovasc Ultrasound 15(1):22 28899401 10.1186/s12947-017-0111-x PMC5596939

[27] Huang X, Lu G, Cai X, Xue Y, Wang X, Jiang Y et al (2024) Myocardial strain is regulated by cardiac preload in the early stage of sepsis. BMC Cardiovasc Disord 24(1):426 39143461 10.1186/s12872-024-04083-8 PMC11323523

[28] Cavus E, Schneider JN, di Carluccio E, Ziegler A, Haack A, Ojeda F et al (2024) Unrecognized myocardial scar by late-gadolinium-enhancement cardiovascular magnetic resonance: insights from the population-based Hamburg City health study. J Cardiovasc Magn Reson 26(1):101008 38341145 10.1016/j.jocmr.2024.101008 PMC10944257

[29] Le TT, Huang W, Singh GK, Toh DF, Ewe SH, Tang HC et al (2021) Echocardiographic global longitudinal strain is associated with myocardial fibrosis and predicts outcomes in aortic stenosis. Front Cardiovasc Med 8:750016 34859068 10.3389/fcvm.2021.750016 PMC8631398

[30] Oketunbi TJ, Wang J, Ding B, Song X, Li Y, Song H et al (2025) Novel insights into myocardial fibrosis in patients with new onset ST-elevation myocardial infarction following percutaneous coronary intervention through enhanced cardiac magnetic resonance imaging: a prospective cohort study. BMC Cardiovasc Disord 25(1):274 40211110 10.1186/s12872-025-04719-3 PMC11983772

[31] El-Saadi W, Engvall JE, Alfredsson J, Karlsson JE, Martins M, Sederholm S et al (2022) A head-to-head comparison of myocardial strain by fast-strain encoding and feature tracking imaging in acute myocardial infarction. Front Cardiovasc Med 9:949440 35966533 10.3389/fcvm.2022.949440 PMC9366255

[32] Grund FF, Kristensen CB, Myhr KA, Vejlstrup N, Hassager C, Mogelvang R (2021) Layer-specific strain is preload dependent: comparison between speckle-tracking echocardiography and cardiac magnetic resonance feature-tracking. J Am Soc Echocardiogr 34(4):377–387 33421611 10.1016/j.echo.2020.12.024

[33] Giusca S, Steen H, Montenbruck M, Patel AR, Pieske B, Erley J et al (2021) Multi-parametric assessment of left ventricular hypertrophy using late gadolinium enhancement, T1 mapping and strain-encoded cardiovascular magnetic resonance. J Cardiovasc Magn Reson 23(1):92 34247623 10.1186/s12968-021-00775-8 PMC8273957

[34] Abou R, Prihadi EA, Goedemans L, van der Geest R, El Mahdiui M, Schalij MJ et al (2020) Left ventricular mechanical dispersion in ischaemic cardiomyopathy: association with myocardial scar burden and prognostic implications. Eur Heart J Cardiovasc Imaging 21(11):1227–1234 32734280 10.1093/ehjci/jeaa187

[35] Gutman SJ, Kawakami H, Taylor AJ, Marwick TH (2020) The relationship between mechanical dispersion and late gadolinium enhancement in patients with nonischemic cardiomyopathy. JACC Cardiovasc Imaging 13(12):2687–2689 32739376 10.1016/j.jcmg.2020.06.010

[36] Aagaard EN, Lyngbakken MN, Kvisvik B, Berge T, Pervez MO, Ariansen I, et al. (2022) Associations between cardiovascular risk factors, biomarkers, and left ventricular mechanical dispersion: insights from the ACE 1950 Study. Eur Heart J Open.2(2):oeac006. 10.1093/ehjopen/oeac006 PMC9242045 35919126

[37] de Meester de Ravenstein C, Bouzin C, Lazam S, Boulif J, Amzulescu M, Melchior J, et al. (2015) Histological validation of measurement of diffuse interstitial myocardial fibrosis by myocardial extravascular volume fraction from modified look-locker imaging (MOLLI) T1 mapping at 3 T. J Cardiovasc Magn Reson.17:48. 10.1186/s12968-015-0150-0 PMC4464705 26062931

[38] Di Marco A, Brown PF, Bradley J, Nucifora G, Anguera I, Miller CA et al (2022) Extracellular volume fraction improves risk-stratification for ventricular arrhythmias and sudden death in non-ischaemic cardiomyopathy. Eur Heart J. 10.1093/ehjci/jeac142 35877070

[39] Donekal S, Venkatesh BA, Liu YC, Liu CY, Yoneyama K, Wu CO et al (2014) Interstitial fibrosis, left ventricular remodeling, and myocardial mechanical behavior in a population-based multiethnic cohort. Circ Cardiovasc Imaging 7(2):292–302 24550436 10.1161/CIRCIMAGING.113.001073 PMC3992469

[40] Frojdh F, Fridman Y, Bering P, Sayeed A, Maanja M, Niklasson L et al (2020) Extracellular volume and global longitudinal strain both associate with outcomes but correlate minimally. JACC Cardiovasc Imaging 13(11):2343–2354 32563637 10.1016/j.jcmg.2020.04.026

[41] Jin C, Weber J, Singh H, Gliganic K, Cao JJ (2021) The association of reduced left ventricular strains with increased extracellular volume and their collective impact on clinical outcomes. J Cardiovasc Magn Reson 23(1):93 34218790 10.1186/s12968-021-00776-7 PMC8256505

[42] Sardanelli F, Schiaffino S, Zanardo M, Secchi F, Cannao PM, Ambrogi F et al (2019) Point estimate and reference normality interval of MRI-derived myocardial extracellular volume in healthy subjects: a systematic review and meta-analysis. Eur Radiol 29(12):6620–6633 31049734 10.1007/s00330-019-06185-w

[43] Thavendiranathan P, Shalmon T, Fan CS, Houbois C, Amir E, Thevakumaran Y et al (2023) Comprehensive cardiovascular magnetic resonance tissue characterization and cardiotoxicity in women with breast cancer. JAMA Cardiol 8(6):524–534 37043251 10.1001/jamacardio.2023.0494 PMC10099158

[44] Kammerlander AA, Marzluf BA, Zotter-Tufaro C, Aschauer S, Duca F, Bachmann A et al (2016) T1 Mapping by CMR imaging: from histological validation to clinical implication. JACC Cardiovasc Imaging 9(1):14–23 26684970 10.1016/j.jcmg.2015.11.002

[45] Duca F, Kammerlander AA, Zotter-Tufaro C, Aschauer S, Schwaiger ML, Marzluf BA, et al. (2016) Interstitial fibrosis, functional status, and outcomes in heart failure with preserved ejection fraction: insights from a prospective cardiac magnetic resonance imaging study. Circ Cardiovasc Imaging. 9(12). 10.1161/CIRCIMAGING.116.005277 27974408

[46] Diao KY, Yang ZG, Xu HY, Liu X, Zhang Q, Shi K et al (2016) Histologic validation of myocardial fibrosis measured by T1 mapping: a systematic review and meta-analysis. J Cardiovasc Magn Reson 18(1):92 27955698 10.1186/s12968-016-0313-7 PMC5154013

