# Supplementary material for: Myocardial fibrosis is associated with worse left ventricular strain and synchrony assessed by novel CMR imaging in the general population: data from the Akershus Cardiac examination 1950 study
Source: Int J Cardiovasc Imaging. 2026 Jun 19;42(9):1801–15. doi: 10.1007/s10554-026-03746-1 (PMC13582216; doi:10.1007/s10554-026-03746-1)
Supplement: Supplementary file 1 [file 10554_2026_3746_MOESM1_ESM.docx]

**Myocardial fibrosis is associated with worse left ventricular strain and synchrony assessed by novel CMR imaging in the general population:**

**Data from the Akershus Cardiac Examination 1950 Study**

Thakshani Wimalanathan^1,2^, Joanna Sulkowska^1, 2^,Aklilu Woldegabriel Melles^1^, Michael F. Paus^2,3^, Julia B. Skranes^2^, Trygve Berge^4,^ Arnljot Tveit^4,5^, Helge Røsjø^2,6^, Torbjørn Omland^2,3^, Magnus N. Lyngbakken^2,3^, Siri Lagethon Heck^1,2^

^1^Department of Diagnostic Imaging, Division of Diagnostics and Technology, Akershus University Hospital, Lørenskog, Norway

^2^K.G. Jebsen Center for Cardiac Biomarkers, Institute of Clinical Medicine, Faculty of Medicine, University of Oslo, Oslo, Norway

^3^Department of Cardiology, Division of Medicine, Akershus University Hospital, Lørenskog, Norway

^4^Department of Medical Research, Bærum Hospital, Vestre Viken Hospital Trust, Gjettum, Norway

^5^Institute of Clinical Medicine, Faculty of Medicine, University of Oslo, Oslo, Norway

^6^Akershus Clinical Research Center (ACR), Division of Research and Innovation, Akershus University Hospital, Lørenskog, Norway

**List of abbreviations:**

fSENC, Fast Strain-ENCoded imaging

**Supplemental material**

**Methods**

*Fast Strain-Encoded (fSENC) imaging*

Three long axis and three short axis SENC images were used to obtain strain. The following imaging parameters were used: TE = 0.712 ms; flip angle = 30**°**, slice thickness = 10mm. TR was 12.16 ms for 3- and 4ch and 13.15 ms for 2ch and short axis fSENC. Strain encoding throughout the cardiac cycle was visualized in the source images using an automatic tissue tracking algorithm with color coding. Circumferential strain was extracted from 3 long-axis views and longitudinal strain was extracted from the 3 short-axis images by drawing endo- and epicardial borders at the end-systolic cardiac phase, excluding papillary muscles. Contours were reviewed and corrected if necessary. Longitudinal strain was reported for 16 segments and circumferential strain for 21 segments of the left ventricle.
**Supplemental tables**

**Table S1.**

| **Association between myocardial fibrosis and strain above gender specific upper quartile** | | | | |
| --- | --- | --- | --- | --- |
| Odds ratio from logistic regression analyses | | | | |
|  | **Model 1**  **OR (95% CI)** | **Model 2**  **OR (95% CI)** | **Model 3**  **OR (95% CI)** | **Model 4**  **OR (95% CI)** |
| GLS**^a^** | | | | |
| LGE presence | 3.55 (1.68, 7.51)^†^ | 3.77 (1.74, 8.17)^†^ | 3.95 (1.71, 9.12)^†^ | 3.56 (1.51, 8.42)^†^ |
| LGE, gram | 1.23 (1.05,1.43)^†^ | 1.27 (1.07, 1.51)^†^ | 1.25 (1.05, 1.48)* | 1.22 (1.03, 1.44)* |
| ECV | 1.04 (0.89, 1.21) | 1.05 (0.89, 1.24) | 1.08 (0.90, 1.29) | 1.05 (0.88, 1.27) |
| GCS**^b^** | | | | |
| LGE presence | 3.06 (1.45, 6.49)^†^ | 3.06 (1.42, 6.57)^†^ | 3.66 (1.56, 8.59)^†^ | 3.32 (1.28, 8.62)* |
| LGE, gram | 1.16 (1.02, 1.33)* | 1.15 (1.02, 1.31)* | 1.14 (0.99, 1.31) | 1.08 (0.94, 1.25) |
| ECV | 0.95 (0.81-1.11) | 0.94 (0.79-1.11) | 0.99 (0.83-1.18) | 0.89 (0.73, 1.08) |
| Abbreviations: CI, confidence interval; ECV, extracellular volume; GCS, global circumferential strain; GLS, global longitudinal strain; LGE, late gadolinium enhancement; OR, odds ratio  ^*^*P* < 0.05, ^†^*P* < 0.01, ^‡^*P* < 0.001  **^a^**Absolute value of GLS dichotomized at sex-specific lower quartile (16.3 for women and 15.5 for men)  **^b^**Absolute value of GCS dichotomized at sex-specific lower quartile (16.7 for women and 15.3 for men)  Model 1, unadjusted; Model 2, adjusted for age and sex; Model 3, adjusted for age, sex, BMI, CRP, eGFR, hypertension, diabetes and smoking status; Model 4, adjusted for model 3 and LVEDVi. | | | | |

**Table S2**

| **Association between myocardial fibrosis and fSENC % normal segments < 80%** | | | | |
| --- | --- | --- | --- | --- |
| Odds ratio from logistic regression analyses | | | | |
|  | **Model 1**  **OR (95% CI)** | **Model 2**  **OR (95% CI)** | **Model 3**  **OR (95% CI)** | **Model 4**  **OR (95% CI)** |
| % normal segments < 80 | | | | |
| LGE presence | 1.55 (0.63, 3.79) | 1.23 (0.47, 3.19) | 1.22 (0.43, 3.50) | 1.34 (0.45. 3.97) |
| LGE, gram | 1.36 (0.92, 2.01) | 1.27 (0.86, 1.86) | 1.26 (0.81, 1.97) | 1.30 (0.83, 2.03) |
| ECV | 0.81 (0.68, 0.96)* | 0.85 (0.71, 1.05) | 0.87 (0.70, 1.08) | 0.88 (0.71, 1.09) |
| Abbreviations: CI, confidence interval; ECV, extracellular volume; fSENC, fast strain-encoded; LGE, late gadolinium enhancement; OR, odds ratio  ^*^*P* < 0.05, ^†^*P* < 0.01, ^‡^*P* < 0.001  Model 1, unadjusted; Model 2, adjusted for age and sex; Model 3, adjusted for age, sex, BMI, CRP, eGFR, hypertension, diabetes and smoking status; Model 4, adjusted for model 3 and LVEDVi. | | | | |

**Table S3**

| **Association of LGE presence/volume and ECV with mechanical dispersion** | | | | |
| --- | --- | --- | --- | --- |
| Beta coefficients from linear regression analyses | | | | **P-value**  **for nonlinearity^a^** |
|  | **Model 1**  **B (95% CI)** | **Model 2**  **B (95% CI)** | **Model 3**  **B (95% CI)** |  |
| MD | | | |  |
| LGE presence | 0.17 (0.34, 0.30)* | 0.14 (0.18, 0.27) ^*^ | 0.13 (-0.004, 0.26) | Not tested^b^ |
| LGE, gram | 0.02 (-0.001, 0.04) | 0.02 (-0.004, 0.04) | 0.02 (-0.005, 0.04) | Not tested^b^ |
| ECV | 0.006 (-0.20, 0.03) | 0.20 (-0.005, 0.04) | 0.02 (-0.02, 0.05) | 1.0 |
| Abbreviations: B, beta coefficient; CI, confidence interval; ECV, extracellular volume; LGE, late gadolinium enhancement; MD, mechanical dispersion  ^*^*P* < 0.05, ^†^*P* < 0.01, ^‡^*P* < 0.001  ^a^P-value for comparison of fully adjusted linear regression analysis with spline regression analysis with 3 knots with Likelihood Ratio Test.  ^b^Spline analyses were not performed due to an insufficient number of participants with detectable LGE  Model 1, unadjusted; Model 2, adjusted for age and sex; Model 3, adjusted for age, sex, BMI, CRP, eGFR, hypertension, diabetes and smoking status. | | | | |

**Table S4**

| **Regional distribution of focal scars** | | | | | | |
| --- | --- | --- | --- | --- | --- | --- |
|  | LGE type  N | Septum  N | Anterior wall N | Lateral wall N | Inferior wall N | Several regions N |
| All | 45 | 16 | 9 | 29 | 18 | 19 |
| Ischemic scar only | 14 | 5 | 4 | 9 | 6 | 7 |
| Nonischemic only | 27 | 7 | 3 | 16 | 9 | 8 |
| Both scar types | 4 | 4 | 2 | 4 | 3 | 4 |
| Abbreviations: LGE, late gadolinium enhancement | | | | | | |

**Table S5 Cut-off values for dichotomization**

| **Cut-off value of dichotomized outcomes** | | | | |
| --- | --- | --- | --- | --- |
|  | **N** | **Cut-off value** | |  |
|  |  | **Female** | **Male** | **Worst quartile (N)** |
| **Lower quartile GLS** | 185 | 16.3 | 15.5 | 46 |
| **Lower quartile GCS** | 185 | 16.7 | 15.3 | 46 |
| **Upper quartile MD** | 185 | 93.4 | 111.5 | 47 |
| Absolute strain values were dichotomized at the sex-specific 25^th^ percentile, and MD at the sex-specific 75^th^ percentile  Abbreviations: GCS, global circumferential strain; GLS, global longitudinal strain; MD, mechanical dispersion | | | | |

**Table S6 Intraobserver variability of strain measurements**

| **Strain measurement** | **ICC (95% CI)** |
| --- | --- |
| GLS from CMR-FT | 0.91 (0.79-0.96) |
| GSC from CMR-FT | 0.98 (0.95-0.99) |
| % normal myocardium from fSENC | 0.87 (0.69-0.95) |
| GLS from fSENC | 0.97 (0.93-0.99) |
| GSC from fSENC | 0.90 (0.78-0.96) |
| Abbreviations: CI, confidence interval, GCS, global circumferential strain; GLS, global longitudinal strain; fSENC, fast strain-encoded; ICC, intraclass correlation coefficient | |

**Supplemental Figures**

**Figure S1**

In fSENC analyses, longitudinal strain is evaluated from three short axis images that together cover 16 left ventricular segments , whereas circumferential strain is derived from three long axis views (2‑, 3‑, and 4‑chamber), each covering seven segments

Ch, chamber; fSENC; fast strain encoded; SA, short axis

**Figure S2**

Histogram of GLS measured in %. Bars represent frequency. GLS, global longitudinal strain.

**Figure S3**

Histogram of GCS measured in %. Bars represent frequency.

GCS, global circumferential strain.

**Figure S4**

Histogram of MD measured ms % adjusted for heart rate. Bars represent frequency.

MD, mechanical dispersion

**Figure S5**

Histogram of fSENC % normal myocardium. Bars represent frequency. FSENC, fast strain-encoded.

**Figure S6 Sensitivity analyses**

c

b

a

Nonlinear association between ECV and GLS (a), GCS (b) and %normal myocardium (c). Knots are placed at ECV 27 % as well as at the 10^th^ and 90^th^ percentile.

**Figure S7 Sensitivity analyses**

c

a

b

Nonlinear association between ECV and GLS (a), GCS (b) and %normal myocardium (c). Knots are placed at ECV 25 % as well as at the 10^th^ and 90^th^ percentile
